# Progress and Applications of Plant Growth-Promoting Bacteria in Salt Tolerance of Crops

**DOI:** 10.3390/ijms23137036

**Published:** 2022-06-24

**Authors:** Yaru Gao, Hong Zou, Baoshan Wang, Fang Yuan

**Affiliations:** Shandong Provincial Key Laboratory of Plant Stress, College of Life Sciences, Shandong Normal University, Ji’nan 250014, China; gyr990108@163.com (Y.G.); zh20040106@163.com (H.Z.)

**Keywords:** salt stress, endosymbiotic bacteria, exosymbiotic bacteria, plant growth-promoting bacteria, PGPR, PGPEB

## Abstract

Saline soils are a major challenge in agriculture, and salinization is increasing worldwide due to climate change and destructive agricultural practices. Excessive amounts of salt in soils cause imbalances in ion distribution, physiological dehydration, and oxidative stress in plants. Breeding and genetic engineering methods to improve plant salt tolerance and the better use of saline soils are being explored; however, these approaches can take decades to accomplish. A shorter-term approach to improve plant salt tolerance is to be inoculated with bacteria with high salt tolerance or adjusting the balance of bacteria in the rhizosphere, including endosymbiotic bacteria (living in roots or forming a symbiont) and exosymbiotic bacteria (living on roots). Rhizosphere bacteria promote plant growth and alleviate salt stress by providing minerals (such as nitrogen, phosphate, and potassium) and hormones (including auxin, cytokinin, and abscisic acid) or by reducing ethylene production. Plant growth-promoting rhizosphere bacteria are a promising tool to restore agricultural lands and improve plant growth in saline soils. In this review, we summarize the mechanisms of plant growth-promoting bacteria under salt stress and their applications for improving plant salt tolerance to provide a theoretical basis for further use in agricultural systems.

## 1. Introduction

Soil salinization is a growing challenge for agriculture. Poor soil stewardship and irrigation practices, as well as the large-scale use of chemical fertilizer, have exacerbated soil salinity problems worldwide, steadily reducing the amount of arable land [1]. Currently, there are more than 800 million hectares of saline soil worldwide, and 20% of irrigated soil is affected by salinity [2,3]. In China, approximately 3.6107 ha of land is considered saline soil, accounting for approximately 4.88% of the total farmland [4]. Excessive levels of salt in the soil inhibit crop growth and reduce yield; therefore, strategies for improving plant salt tolerance to better use saline soils are urgently needed.

High salt concentrations within plant tissue disrupt the cellular ion balance, leading to reactive oxygen species (ROS) production and Na^+^ and Cl^−^ ion accumulation [5]. Excessive levels of ROS (oxygen radicals, superoxide, and hydrogen peroxide) destroy cellular structures and biomolecules. For example, ROS degrade chlorophyll and peroxidize lipids, which reduces photosynthetic activity, damages cell membranes, and eventually induces cell death [6]. Na^+^ and Cl^−^ ions interfere with enzyme function and physiological processes. For instance, Na^+^ and Cl^−^ ions interfere with stomatal opening and closing, resulting in osmotic stress and reduced photosynthesis [7,8]. Furthermore, high concentrations of Cl^−^ ions inhibit nitrate reductase activity, which leads to nutrient imbalances [9]. Salt stress elevates ethylene levels in plants, which causes premature senescence and defoliation [10].

Several strategies for improving saline soils have been tested, including chemical and physical methods and the structural engineering of land for ecological restoration [11]. Examples include the improved surface runoff on the saline soils of the Yellow River Delta in China based on the auxiliary infiltration model of saline drainage engineering [12]. Using a network of natural sedimentation and evaporation terrace drainage ditches established the centralized transfer of excess salt, so as to achieve the discharge of soil salts [13]. However, these structural engineering measures treat the symptoms but not the root cause. Some unreasonable measures such as excessive drainage and freshwater pressure can also cause secondary soil salinization [14]. Biological and organic products offer an environmentally friendly approach to soil restoration. Amendments such as biochar can be used to improve the physicochemical properties of soil. Amendments such as biochar can be used to improve soil physicochemical properties. Biochar application has been used to repair depleted and saline–alkali soils [15]. Wu [16] reported that biochar increases the organic matter content and soil microbial activity, and thereby the nutritional status of saline soil.

Another biological restoration approach is to introduce genes into crops from naturally salt-tolerant plants such as halophytes. However, this is a time-consuming and costly process. Halophytes accumulate and discharge salt through their roots and/or leaves [17]. Therefore, halophytes are excellent models for studying crop salt tolerance and are a rich source of salt tolerance genes [18]. How to make crops not only thrive but also improve yield in saline environments is a hot research topic.

Soil microorganisms offer yet another approach for soil restoration and improving plant salt tolerance. Symbiotic bacteria promote plant growth and alleviate salt stress by providing minerals (such as nitrogen, phosphate, and potassium) and hormones (including auxin, cytokinin, and abscisic acid) or by reducing ethylene production. There are two types of plant growth-promoting bacteria: exosymbiotic bacteria and endosymbiotic bacteria. The former are associated with the outside of roots in the rhizosphere, also known as plant growth-promoting rhizobacteria (PGPR) [19]. The latter reside within roots or form a symbiont and are called plant growth-promoting endophytic bacteria (PGPEB). Salt-tolerant plants and bacteria co-evolved survival strategies to adapt to high-salt environments [20,21,22,23].

The current review summarizes the mechanism of plant growth-promoting bacteria under salt stress and its application in improving plant salt tolerance in order to provide a theoretical basis for the further application of plant growth-promoting bacteria in agricultural systems.

## 2. Summary of Plant Growth-Promoting Bacteria Isolation Procedures

**Figure 1:**
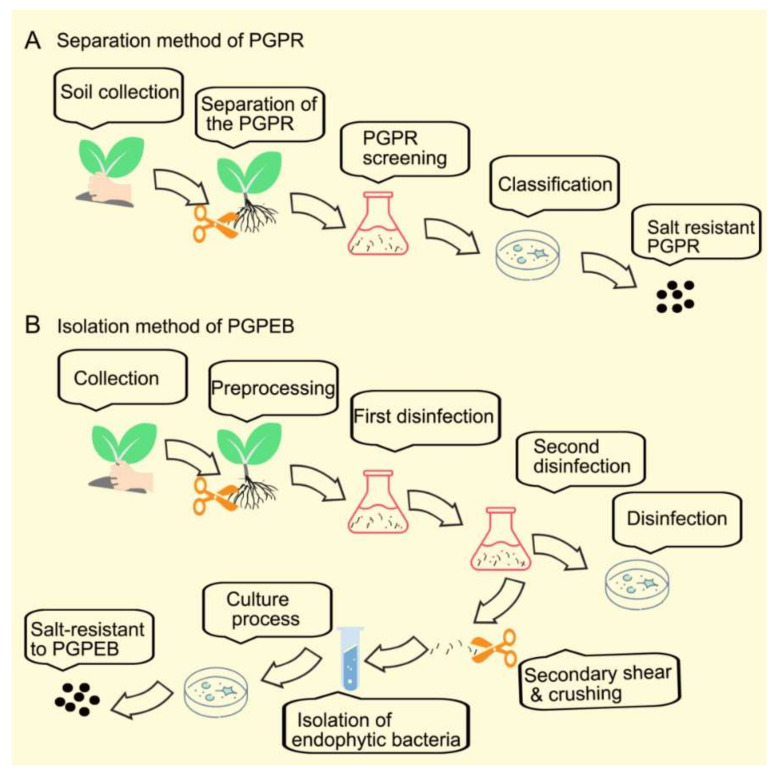
Isolation summary of PGPR (**A**) and PGPEB (**B**).

At present, the use of fertilizer is causing great pressure on the environment in terms of environmental pollution and economic cost. Researchers have started to isolate and screen candidate promoting strains from some crops (such as wheat (*Triticum aestivum*) and rice (*Oryza sativa*)). There are many characteristics and commonalities of the separation method in PGPR and PGPEB as described below. To isolate PGPR, the rhizosphere soil is washed from the roots. Different specialized media for bacterial isolation, culture, and purification are prepared (Figure 1). NaCl is added to the medium to screen for salt-tolerant bacteria. To characterize the bacteria, vernier calipers are used to measure the colony diameter, and the salt tolerance threshold and pH tolerance are determined. The strains are then identified by sequence analysis. The gene sequences of related species are selected in the NCBI database to determine the taxonomy of the strains.

As for PGPEB, the roots are rinsed, surface disinfected, cut into small sections, and homogenized. The homogenate is then spread on agar media to isolate the PGPEB under saline conditions. After the dilution and further culture of the supernatant, colonies are purified and sequences are compared for species identification.

## 3. Growth-Promoting Bacteria Related to Salt Stress Currently Found in Plants

Salt affects the physicochemical and chemical properties and biological characteristics of the soil [24], which adversely affects plant growth, development, and reproduction. Plant symbionts alleviate the effects of salt stress by promoting seed germination, organ differentiation, biomass accumulation, and nutrient absorption; regulating plant hormone homeostasis [25,26]; inducing the antioxidant system; and maintaining ion homeostasis. Different plant growth-promoting bacteria have different regulatory mechanisms and different protective effects in monocots and dicots.

Table 1 illustrates the different mechanisms of action of different plant growth-promoting bacteria on monocots and dicots. Five monocotyledons and twenty-two dicotyledons related to PGPR were introduced. Many dicot crops have been reported to have enhanced salt tolerance after inoculation with plant growth-promoting bacteria, such as *Arabidopsis thaliana* (Arabidopsis), common bean (*Phaseolus vulgaris*), and radish (*Raphanus sativus*). Five monocot crops were reported to have better salt tolerance after being treated with plant growth-promoting bacteria via enhanced 1-aminocyclopropane-1-carboxylic acid (ACC) deaminase activity, increased antioxidant enzyme activity, and improved osmotic regulation. Among the twenty-two dicotyledons, five plants enhanced their salt tolerance by increasing the IAA content through PGPR. Eight crops enhanced their salt tolerance through PGPR by enhancing ACC deaminase synthesis.

**Table 1:** Interaction of plant growth-promoting bacteria (PGPR) under stress and their beneficial effects.

Plant Species	PGPR Species	Effect or Mechanism	Reference
*Avena sativa*	*Klebsiella* sp.	Regulating ion contents and proline levels	[27]
Barley (*Hordeum vulgare*)	*Curtobacterium* sp.	Regulating proline content	[28]
	*Hartmannibacter diazotrophicus*	Enhancing ACC deaminase activity	[29]
Maize (*Zea mays*)	*Bacillus atrophaeus*	Relieving salt stress	[30]
	*Azotobacter* sp.	Promoting nutrient absorption in plants	[31]
	*Bacillus amyloliquefaciens SQR9*	Enhancing antioxidant enzyme activity and increasing the expression of salt-stress-response genes	[32]
	*Bacillus* sp.	Increasing enzyme activities and proline and soluble sugar contents under salt stress; regulating ACC deaminase activity	[33,34]
	*Enterobacter cloacae PM23*	Modulating plant physiology, antioxidant defense, compatible solute accumulation, and bio-surfactant-producing genes	[35]
	*Geobacillus* sp.	Regulating proline content	[36]
	*Pseudomonas* sp.	Enhancing proline and IAA content and EPS production	[37]
	*Rhizobium* sp.	Regulating pigment biosynthesis	[38]
Rice (*Oryza sativa*)	*Bacillus aryabhattai*, *Achromobacter denitrificans*, and *Ochrobactrum intermedium*	Relieving salt stress	[39,40]
	*Bacillus pumilus* and *Pseudomonas pseudoalcaligenes*	Increasing the absorption of nutrients	[41]
	*Enterobacter* sp.	Reducing ethylene production	[42]
	*Glutamicibacter* sp. *YD01*	Adjusting ethylene contents	[43]
	*Micrococcus* sp.	Increasing IAA levels	[44]
	*Rhizobacteria pseudomonas*	Regulating ACC deaminase activity	[40]
Wheat (*Triticum aestivum*)	*Aeromonas* sp.	Regulating ethylene content and alleviating salt stress	[45]
	*Arthrobacter* sp.	Maintaining plant nutrient absorption	[46]
	*Bacillus* sp.	Regulating ACC deaminase activity	[47,48]
	*Enterobacter* sp.	Reducing ethylene production	[49]
	*Microbacterium* sp.	Regulating K^+^ content	[45]
	*Planococcus rifietoensis*	Regulating phosphate production and ACC deaminase activity	[50]
	*Pseudomonas fluorescence*, *Bacillus pumilus*, and *Exiguobacterium aurantiacum*	Adjusting osmotic substances	[51]
	*Klebsiella* sp.	Regulating ion contents, proline levels, and antioxidant enzyme activity	[52,53,54]
	*Serratia* sp.	Production of exopolysaccharides	[49,55]
	*Serratia marcescens CDP-13*	Enhancing ACC deaminase activity and reducing salt-induced oxidative damage	[55]
*Arabidopsis thaliana*	*A* *cillus atropheus*	Relieving salt stress	[30]
	*Bacillus* sp.	Adjusting ACC deaminase activity	[56]
	*Enterobacter* sp.	Reducing ethylene production by promoting ACC deaminase activity	[37,57]
	*Enterobacter* sp. *SA187*	Enhancing sulfur metabolism	[58]
	*Micrococcus* sp.	Increasing IAA levels	[44]
*Arachis hypogaea* L.	*Brachybacterium* sp.	Regulating K^+^ content	[59]
	*Brevibacterium* sp.	Regulating K^+^ content	[59]
	*Haererohalobacter* sp.	Regulating K^+^ content	[59]
	*Ochrobactrum* sp.	Regulating IAA levels and ACC deaminase activity	[60]
	*Stenotrophomonas maltophilia BJ01*	Modulating physiology and biochemical activities	[61]
*Casuarina obesa* (*Miq.*)	*Pantoea agglomerans* and *Bacillus* sp.	Increasing total chlorophyll production and proline accumulation	[62]
*Codonopsis pilosula*	*Bacillus* sp.	Adjusting ACC deaminase activity	[63]
Common bean (*Phaseolus vulgaris*)	*Aneurinibacillus Aneurinilyticus* and *Paenibacillus* sp.	Adjusting ACC deaminase activity	[64]
Cucumber (*Cucumis sativus*)	*Bacillus* sp.	Adjusting ACC deaminase activity	[65]
	*Burkholdera* sp.	Maintaining the water balance and regulating photosynthetic pigment content	[8]
Strawberry (*Fragaria ananassa*)	*Kocuria* sp.	Maintaining phosphate	[66]
Cotton (*Gossypium hirsutum*)	*Pseudomonas* sp.	Enhancing proline, IAA, and EPS content production	[67]
*Helianthus Annuus* L.	*Azospirillum* sp.	Regulating chlorophyll content and improving photosynthesis	[68]
*Lens esculenta*	*Oceanobacillus* sp.	Production of exopolysaccharides	[69]
Lettuce (*Lactuca sativa*)	*Pseudomonas mendocina Palleroni,* arbuscular mycorrhizal (AM) fungus	Improving antioxidase activity	[70]
*Limonium sinense*	*Streptomyces* sp.	Enhancing proline production	[71]
*Medicago cilitaris*	*Sinorhizobium* sp.	Promoting proline production	[72]
*Mentha arvensis*	*Exiguobacterium* sp.	Production of exopolysaccharides	[73]
*Pistacia vera* L.	*Arthrobacter endophyticus,Zobellella denitrificans* and *Staphylococcus sciuri*	Improving photosynthesis	[74]
Pea (*Pisum sativum*)	*Arthrobacter* sp.	Increasing nutrient uptake	[75]
	*Rhizobium* sp.	Regulating pigment synthesis	[75]
	*Variovorax* sp.	Enhancing ACC deaminase activity	[76]
Radish (*Raphanus* *sativus*)	*Lactobacillus* sp., *P. putida*, and *Azotobacter* *chroococcum*	Mitigating salinity stress at the time of germination	[77]
*Sesuvium portulacastrum*	*Halobacillus* sp.	Production of ammonia and cyanide (HCN)	[78]
*Silybum marianum*	*Pseudomonas* sp.	Enhancing proline and IAA content and EPS production	[79]
Soybean (*Glycine max*)	*Arthrobacter woluwensis*, *Microbacterium oxydans*, *Arthrobacter aurescens, Bacillus megaterium*, and *Bacillus aryabhattai*	Maintaining osmotic balance and regulating salt tolerance	[80]
Tomato (*Solanum lycopersicum*)	*Achromobacter* sp.	Adjusting ethylene content	[81]
	*Enterobacter* sp.	Reducing ethylene production	[57]
	*Growth-promoting rhizobacteria*	Relieving water stress and increasing K^+^ absorption	[82]
	*Leclercia adecarboxylata MO1*	Promoting the production of IAA and ACC	[83]
	*Sphingomonas* sp.	Exopolysaccharides and proline production	[84,85]
*Vigna radiata* L.	*Enterococcus* sp.	Reducing sodium uptake	[86]
	*Pantoea* sp.	Improving ACC deaminase activity	[86]
	*Rhizobium* sp.	Increasing chlorophyll and photosynthesis	[87]

## 4. The Mechanism of PGPR in Improving Stress Tolerance

Salinity stress adversely affects plant morphology, physiology, and biochemical functions. Some plants (especially halophytes) accumulate salt in the xylem and extrude it through the leaves, while others have evolved special structures (salt glands) to excrete the salt, which is removed by external forces such as wind or water. Yuan [88] found that the unique root microbiota of Suaeda salsa not only improve its adaptability to saline soils, but also improve other non-halophytes such as cucumber and rice. Endophytes in plant tissues help plants resist drought stress through various chemical substances (abscisic acid, indole-3-acetic acid, ACC deaminase, and various volatile compounds) released by themselves [89]. Although both exogenous PGPR and endogenous PGPR can improve the stress response of plants, the living environment of endophytes is not affected by soil pH and other bacteria [90] and their mechanisms are different.

**Figure 2:**
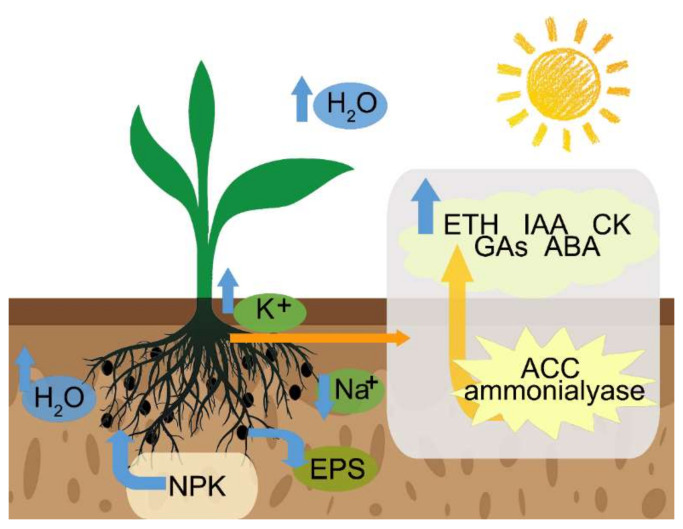
Mechanism of PGPR in salt stress alleviation. Black circles surrounding the roots represent the PGPR. Under salt stress, plants reduce transpiration and water loss by increasing K^+^ absorption and reducing Na^+^ absorption, thus alleviating osmotic stress and ion stress; PGPR promote plant growth by increasing nutrient absorption; meanwhile, PGPR regulate hormone production (IAA, GAs, CK, and ABA) and ACC deaminase activity to alleviate salt stress. Exopolysaccharides (EPSs) are homologous or heteropolysaccharides produced by rhizosphere bacteria. EPSs bind soil particles into aggregates to form a closed substrate that increases root adhesion to the soil (RAS/RT) in each root tissue, giving protection from environmental fluctuations. Protective EPS capsules have a strong water retention capacity, protecting plants from desiccation under salt stress, as well as help plants to absorb nutrients.

A model describing how PGPR enhances plant salt tolerance is shown in Figure 2. PGPR improves the salt tolerance of plants through the following mechanisms: (1) inducing the antioxidant system; (2) maintaining the water balance within the plant, releasing bound phosphorus and potassium from the soil, chelating iron, and fixing atmospheric nitrogen [90]; (3) selectively absorbing K^+^ and excluding Na^+^ to maintain a high K^+^/Na^+^ ratio [91]; (4) using PGPR release of exopolysaccharides (EPSs) [92] as the formation of protective biofilms reduces the toxicity of Na^+^; (5) maintaining plant hormone levels [93,94]; and (6) increasing osmotic regulatory substances.

### 4.1. Inducing the Antioxidant System

Salt stress induces ROS production (including superoxide radical (O_2_^−^), hydroxyl radical (OH^−^), and hydrogen peroxide (H_2_O_2_)), which damages DNA, alters the redox status, perturbs protein formation, degrades membrane proteins, peroxidizes lipids, reduces membrane fluidity, and interferes with enzyme activity, resulting in cell damage and potentially cell death. Under these conditions, enzymatic antioxidant systems (such as superoxide dismutase (SOD), catalase (CAT), and ascorbate peroxidase (APX)) and non-enzymatic antioxidants (such as glutathione (GSH) and ascorbate) play important roles in neutralizing ROS to protect plant cells from oxidative stress. PGPR induce the plant’s antioxidant system to protect plants from oxidative stress. Salt stress induces the adaptive response mechanisms, including the accumulation of compatible substances (including organic matter and inorganic substances, such as proline and soluble sugars) and reducing membrane hydraulic potential to reduce osmotic stress [95,96]. PGPR inoculation in potato grown under salt stress conditions enhanced APX, SOD, CAT, and glutathione reductase activities [97]. *Azospirillum lipoferum FK1* inoculation enhanced nutrient absorption and the levels of antioxidant enzymes [98]. *Trichoderma*, *Pseudomonas*, and their combination inoculation increased peroxidase (POD), APX, SOD, and CAT activities to alleviate salt stress [99]. These results indicate that PGPR help protect plants from oxidative stress.

### 4.2. Maintaining the Water Balance and Access to Nutrients

Cell hydration is critical for plant physiological and metabolic processes and for plant growth. The potential water gradient in the xylem guides evapotranspiration from root to leaf, preventing an imbalance between transpiration rates in aboveground organs and soil water absorption. Under osmotic stress, photosynthesis decreases and plant growth slows. The inoculation of beneficial bacteria into pepper roots enhances root systems, thereby increasing the plant’s ability to absorb water from the surrounding environment [100]. The proportion of intracellular aquaporins determines the hydraulic conductivity on the root surface, thus determining the plant absorption of saline soil water [101]. Plasma membrane intrinsic proteins (PIPs) are important aquaporins in plants and enable adaptation to changing environmental conditions [102]. The gene expression analysis of maize (*Zea mays*) roots inoculated with *Bacillus megaterium* and *Pantoea agglomerans* revealed that the upregulation of *ZmPIP2* and *ZmPIP1-1* resulted in increased water uptake under salt stress conditions [103]. These studies suggest that PGPR promote plant tolerance to osmotic stress.

Bacteria residing on the root surface help plants absorb water and nutrients through nitrogen fixation, phosphate dissolution, and siderophore production [104,105]. Nitrogen is an essential nutrient and is often exogenously applied in large quantities. However, inorganic fertilizers often alter soil structure and, thus, the soil microbiota composition [106]. The relationship between nitrogen-fixing rhizobia and legume roots is well studied. In this symbiotic relationship, the rhizobia provide nitrogen to legumes, yielding reduced carbon and creating a suitable environment for nitrogenase activity [107]. All stages of nitrogen fixation in legumes are sensitive to salinity, and improving salt tolerance in diazotrophs, such as *Azospira*, increases the yield of various cereal crops [108,109].

Under salt conditions, PGPR can promote plant growth and its absorption and utilization of mineral nutrients. Phosphorus is an essential nutrient but primarily exists in an insoluble form in soil, making phosphorus deficiency a common problem in crop production. Phosphate-solubilizing microorganisms (PSMs) convert phosphate into an easily accessible, soluble form for use by plants [107]. When plants are exposed to salt stress, Phosphate-solubilizing bacteria (PSB) include strains of *Arthrobacter Pseudomonas*, *Bacillus* and *Rhizobium* which secrete acidic substances [110,111] to acidify the soil and improve phosphorus utilization through ion chelation and ion exchange [112]. Furthermore, the soluble phosphorus produced by PSB were combined with heavy metals to become insoluble substances, thereby reducing the content of heavy metals in the soil [113]. Several salt-tolerant PGPR have been identified. Zhu [114] isolated *Kushneria* sp. *YCWA18* from Copper East Coast Bridge salt, which has a high phosphate solubilization capacity and grows normally on a solid medium containing 20% NaCl. Iron is a trace element in plants but is critical for many biochemical processes including photosynthesis [115]. Although the iron content in the soil is generally higher than the plant’s iron requirement, plants growing in calcareous soils are more prone to iron deficiency due to the lack of natural iron sources [105,116]. Siderophores are metal chelators produced by PGPR that bind and transport various metals to improve uptake and protect plants from pathogenic bacteria [117,118]. The respiration, photosynthesis and nitrogen fixation in plants are all related to siderophore production. Several siderophore-producing PGPR have been found to be associated with halophytes [119,120,121].

### 4.3. Maintaining Ion Homeostasis

Under salt stress, Na^+^ flows into the roots through the xylem and eventually accumulates on the leaf surface [122]. Na^+^ efflux from plants is difficult because only a small fraction of Na^+^ moves through the phloem to the root, where excessive Na^+^ is toxic to the plant. Excessive aboveground Na^+^ concentrations disturb the activities of respiration and photosynthesis enzymes, increase the Na^+^/K^+^ ratio, and inhibit cytosolic enzymes [123,124]. Salt stress activates Ca^2+^ channels to initiate Ca^2+^ signaling. The Ca^2+^ signal is sensed by calmodulin (CBL4; also called SOS3). Calmodulin forms a complex with CBL-interacting protein kinases (CIPK24; also called SOS2) to phosphorylate SOS1, which is important for maintaining the Na^+^/K^+^ ratio [125,126,127]. PGPR maintains ion homeostasis by increasing the affinity of K^+^ transporters [128] and by restricting the Na^+^ uptake in the root to reduce Na^+^ accumulation in aerial organs [129].

### 4.4. Production of Exopolysaccharides

Exopolysaccharides are homo- or heteropolysaccharides produced by rhizosphere bacteria that improve bacterial survival under adverse conditions. The composition of polysaccharides varies, but all include the monomers glucose, galactose, and mannose and bind to other components such as aminoglycans and urinary sugars to form a capsule-like protective biofilm on the root surface [130]. This biofilm traps excess Na^+^ and inhibits its uptake into the roots [131]. Wheat (*Triticum aestivum*) plants inoculated with *Aeromonas hydrophila* and *Bacillus* accumulated EPS on the roots, which capture Na^+^ and restrict its uptake [45]. Inoculation of *Bacillus subtilis* GB03 into Arabidopsis roots downregulated the genes associated with the ion homeostasis (*HKT1*) of the K ion transporters and reduced the Na^+^ uptake [101,132]. Under salt stress, the inoculation of *Halomonas variabilis (HT1*) and *Planococcus rifietoensis (RT4*) on chickpea (*Cicer arietinum*) stabilized the soil structure and soil aggregates, which improved the chickpea growth [133]. The inoculation of plants with *B. subtilis* improves salt stress tolerance and downregulates the expression of HKT1 transporter genes [132]. Quinoa (*Chenopodium quinoa*) seed inoculated with *Enterobacter* sp. MN17 and *Bacillus* exhibited improved water uptake when grown in high salt (2.34% NaCl) concentrations [134]. The inoculation of *B. subtilis* ssp. and *B. lipois* SM19 significantly reduced the adverse effects of salt stress in wheat [135].

### 4.5. Induction of Plant Hormones

Most PGPR produce IAA, which enhances plant growth under salt stress [25]. Tryptophan in root exudates is converted to IAA by rhizosphere bacteria, which is then absorbed by plant roots [136,137]. Inoculation with *P. stutzeri*, *P. putida*, and *Stenotrophomonas maltophilia* to *Coleus* plants was found to increase IAA, cytokinin (CK), and gibberellic acid (GA) production [26]. PGPR may respond to salt stress by synthesizing CK or altering hormone homeostasis in plants. In addition to promoting growth under salt stress conditions, inoculation with *Pseudomonas* sp. (*P. aurantiaca* and *P. extremorientalis TSAU6* and *TSAU20*) also relieves salt-induced seed dormancy [138]. The ability of PGPR to synthesize CK highlights their importance in stimulating plant growth.

GA regulates cell division, elongation, and root and leaf meristem activities. It also plays an important role in plant development and physiological processes. The PGPR *Azospira* sp. helps produce GA [139]. Abscisic acid (ABA) is a stress-responsive hormone that plays a role in leaf shedding and plant growth. Under water deficit, ABA regulates plant adaptation to stress by activating stress resistance genes. ABA is transferred from root to leaf to control stomatal closure to reduce transpiration on the leaf surface and limit water loss. ABA-producing PGPR may also play an important role in plant–PGPR interactions [25]. PGPR improve plant tolerance to osmotic stress by regulating ABA biosynthesis or translocation [140].

Ethylene, another stress-responsive hormone, increases salt tolerance by negatively regulating root growth and downregulating nitrogen fixation [141]. ACC deaminase is an intracellular enzyme that inhibits ethylene biosynthesis, and can degrade the ethylene precursor ACC, thereby reducing ethylene levels during plant growth and helping to alleviate salt stress. PGPR hinder ethylene biosynthesis by secreting ACC deaminase [142]. ACC deaminase breaks down ACC (an ethylene precursor) into beta-ketone glutaric acid and ammonia, which alters the expression of the ACC oxidase gene involved in ACC synthetase. ACC deaminase-producing strains of *P. fluorescens* and *Enterobacter* spp. significantly improved maize growth under salt stress [143].

Of course, in the process of the plant relief of salt stress, it is often not a single hormone alone, but the result of multiple hormone interactions. There is a synergistic effect between plant hormones, and low concentrations of IAA and GA can promote plant growth to alleviate salt stress; IAA promotes the division of the nucleus, while CK promotes the division of the cytoplasm [144], and the two together complete the division of the nucleus and plastid, thereby promoting plant growth. In addition, ABA adjusts the opening and closing of stomata, thereby adjusting photosynthesis to relieve salt stress [25]; however, high concentrations of IAA can promote the synthesis of ethylene to improve plant salt resistance.

### 4.6. Increasing Osmotic Substances

Increased Na^+^ absorption by plants under salt stress causes osmotic and oxidative stress [145]. Malondialdehyde (MDA) is generated by the lipid peroxidation of the plasma membrane by ROS [146]. PGPR reduced the MDA content under salt stress in several plants: in wheat inoculated with *B. giganossus* [147], maize inoculated with *Kocuria rhizophila* Y1 [148], and canola (*Brassica napus*) inoculated with *E. cloacae* HSNJ4. [147] Furthermore, inoculation with *Azotobacter* sp. increased the free radical scavenging activity in the pennyroyal (*Mentha pulegium* L.) under salt stress [146].

Rhizobia may trigger specific chemical changes in plants—such as changes in total protein, IAA, total sugar, and ethylene content—to improve abiotic stress tolerance, a process known as inducible systemic tolerance [149]. Soluble solutes (sugar and protein) mitigate the lethal effects of salt stress and maintain ion balance in cells [150]. A salt-tolerant *Bacillus* strain that promotes bacterial growth in the rhizosphere improved maize growth and development under drought and saline conditions [151]. Proline has multiple functional roles in response to many abiotic stresses, such as an osmoprotectant and for stabilizing cellular structures and ROS clearance [152]. Pritsh et al. [153] observed that the bacteria belonging to *Bacillus*, *Microbacterium*, *Enterobacter*, *Narnitrophomonas*, *Microbacterium*, and *Acrobacter* increase the proline content in rice (*Oryza sativa*).

## 5. The Role of PGPEB in Alleviating Salt Stress

**Figure 3:**
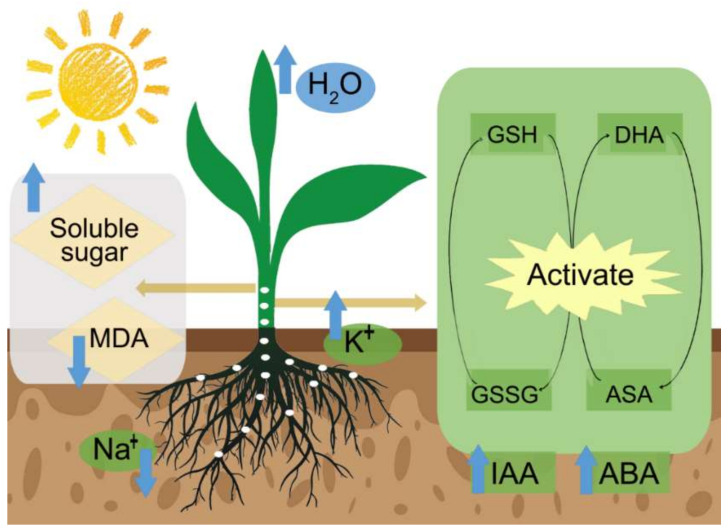
Mechanisms of PGPEB in salt stress alleviation. White dots represent the PGPEB in the plant. Under salt stress, endophytes coregulate the hormone balance, including increasing IAA and ABA contents and activating the ASA-GSH cycle, alleviating osmotic stress by increasing K^+^ and Na^+^ absorption, and alleviating oxidative stress.

Endophytic bacteria are detected in almost all land plants [154]. The endophytic bacteria community structure depends on soil biotic and abiotic factors, host colonization factors, and the ability to survive and compete within host plant tissues. Endophytes interact with plant tissues and participate in various physiological activities. Plants without endophytes have less ability to cope with pathogens and are more susceptible to environmental stresses. Table 2 describes six monocotyledons and nine dicotyledons associated with PGPEB. Seven of the six monocotyledons improved stress resistance by enhancing ACC deaminase synthesis through PGPRB, and seven crops improved stress resistance by increasing the IAA content through PGPEB. Among the nine dicotyledons, seven plants improved stress resistance by enhancing ACC deaminase synthesis through PGPRB, and seven crops improved the stress resistance by increasing the IAA content through PGPEB. PGPEB inoculation increases plant salt tolerance via nitrogen fixation, the modulation of plant hormone levels (auxin, cytokinin, ethylene, and gibberellin), phosphate, iron and potassium solubilization, secondary metabolite synthesis, antibiosis activities against plant pathogens [155], and enhancing photosynthesis (Figure 3).

**Table 2:** Interaction of plant growth-promoting rhizobacteria (PGPEB) under stress and their beneficial effects.

Plant Species	PGPEB Species	Effect or Mechanism	Reference
Cape (*Aloe ferox Mill)*	*Achromobacter xylosoxidans*	Enhancing ACC deaminase activity	[156]
Millet (*Pennisetum glaucum*)	*Bacillus subtilis, Bacillus cereus*, and *Bacillus amyloliquefaciens*	Participating in ACC deaminase synthesis and enhancing IAA content	[157]
Onion (*Allium cepa)*	*Bacillus subtilis, Bacillus megaterium*, and *Burkholderia phytofirmans*	Participating in ACC deaminase synthesis and enhancing IAA content	[158,159]
Rice (*Oryza sativa*)	*Pantoea ananatis*	Enhancing IAA content and siderophore production	[160]
Sugarcane (*Saccharum officinarum*)	*Gluconacetobacter diazotrophicus*	Enhancing IAA content and nitrogen fixation	[161,162]
Wheat *(Triticum aestivum)*	*Paraburkholderia, phytofirmans*, and *Bacillus cabrialessi*	Recovery of nitrogen, phosphorus, and potassium	[163,164]
*Arabidopsis thaliana*	*Serratia proteamaculans Para* and *burkholderia phytofirmans*	Enhancing IAA content and enhancing ACC deaminase activity	[165,166]
*Arachis hypogaea*	*Chryseobacterium indologenes, Enterobacter cloacae, Klebsiella pneumoniae, Pseudomonas aeruginosa*, and *Enterobacter ludwigii*	Nitrogen fixation, enhancing IAA content and ACC deaminase production, siderophore production, and phosphate solubilization	[167]
Cotton (*Gossypium hirsutum*)	*Pantoea* spp., *Empedobacter* spp., *Enterobacter* spp., *Rhizobium* spp., and *Klebsiella* spp.	Adjusting ACC deaminase activity	[168,169]
*Dodonaea viscosa*	*Streptomyces alboniger*, *Bacillus idriensis*, *Pseudomonas taiwanensis*, and *Pseudomonas geniculate*	Siderophore production, phosphate solubilization, enhancing IAA content and ACC deaminase production	[170]
*Helianthus Annuus* L.	*Stentotrophomonas indicatrix*	Enhancing IAA content, phosphate solubilization, siderophore and secondary metabolite synthesis	[171]
Poplar (*Populus*)	*Stenotrophomonas maltophilia*, and *Pseudomonas putida*	Enhancing IAA content and ACC deaminase synthesis	[172]
Potato *(Solanum tuberosum)*	*Klebsiella oxytoca*, *Pseudomonas marginalis*, *Pseudomonas Viridilivida*, *Bacillus endophyticu*, and *Bacillus atrophaeus*	Nitrogen fixation and phosphatase production	[173,174]
Soybean *(Glycine max)*	*Bradyrhizobium japonicum*	Enhancing IAA content and ACC deaminase production, nitrogen fixation	[172,175]
Tomato *(Solanum lycopersicum)*	*Pseudomonas fluorescens* and *Pseudomonas migulae*	Enhancing IAA content and ACC deaminase synthesis	[176,177]

ROS are produced under various stresses, and two systems are involved in ROS scavenging: the enzymatic system and the non-enzymatic system. The endophytic fungus *Piriformospora* improves the antioxidant enzyme activity and salt tolerance of barley (*Hordeum vulgare*) under salt stress [178]. Similarly to PGPR, PGPEB significantly reduce MDA production, as observed with *Streptomyces* inoculation [179]. Endophytic actinomycetes also promote host plant salt tolerance by regulating stomatal aperture. Endophytes also produce biologically active substances and regulate host hormone levels to allow the host to quickly respond to water deficit.

Endophytes promote salt tolerance by reducing the Na^+^ and Cl^−^ content and increasing the aboveground part K^+^ content in the roots. Furthermore, the Na^+^ absorbed by plants mainly accumulates in the roots but is restricted from entering the shoots [180]. Thus, under salinity stress, endophytes change the ion balance of host plants to reduce ion toxicity and alleviate cellular damage. Similar to PGPR, PGPEB enhance plant salt tolerance by increasing IAA levels. For example, some endophytes regulate the auxin content in halophytes and use the antagonism between auxin and ethylene to improve salt tolerance. These halophytic endophytes could be applied to improve salt tolerance in other crops. Tiwari and colleagues [181] demonstrated that wheat plants growing in saline soil had increased fitness when inoculated with IAA-producing rhizosphere bacteria [80]. The ABA is associated with the stomata opening and closing. An endophytic fungus isolated from soybean (*Glycine max*) produces GA, which reduces the plant ABA content [182], thereby more rapidly responding to water loss and accelerating stomatal closure through a range of signaling and hormonal regulatory processes [183].

PGPR and PGPEB play an important role in plant salt tolerance, and most PGPR and PGPEB can both enhance plants’ ability to cope with stresses by increasing plant IAA content, inducing peroxidation systems, maintaining ionic homeostasis, and synthesizing ACC deaminase or enhancing ACC deaminase activity. PGPR can also reduce plant Na^+^ uptake by releasing extracellular polysaccharides to restrict the free flow of soil Na^+^, so as to alleviate the damage of salt stress to plants. In conclusion, PGPR and PGPEB play important roles in plant salt tolerance and can be used with halophytes and non-halophytes to improve saline soils.

## 6. Future Perspectives

### 6.1. Halophytes Can Be Used to Identify Rhizosphere Bacteria

Symbiotic bacteria are beneficial bacteria associated with the rhizosphere and plant roots, and they improve plant salt tolerance. However, the long-term effectiveness and stability of inoculating rhizobia from halophytes onto non-halophyte plants is not well known. Extracting plant growth-promoting bacteria from halophytes and utilizing them in crop production is challenging [184]. Synthesized biopolymer esters have been used to improve bacterial survival and persistence in inoculated crops by slowly releasing the bacteria into the soil. The activity of some halophyte-associated PGPB was maintained and enhanced with biopolymer esters. Meanwhile, the type of iron-friendly complexes can also increase PGPB salt tolerance by the production of iron-chelating substances [185], enabling PGPB to utilize the ferrophilins synthesized by other soil microorganisms. The extraction of plant growth-promoting bacteria from halophytes improves the salt resistance of some non-halophytes. Studies have shown that the isolation and screening of rhizosphere bacteria and root endophytes with two strains (*Halomonas*, *Bacillus*) in the presence of 1% NaCl can promote alfalfa growth, and *Bacillus* has a stronger effect on stem and root biomass [184].

### 6.2. Methods to Improve Symbiont Bacteria Utilization

Enzymes that hydrolyze fungal cell walls are important for plant disease resistance. Studies have shown that in the process of plant pathogen fungus infection host, plant endophyte chitinella (*Chitinophagaceae*) and yellow bacterium (*Flavobacteriaceae*) family members were enriched in plants, and showed enhanced enzyme activity related to fungal cell wall degradation, as well as NRPSs and PKSs encoding secondary metabolites biosynthesis, so as to provide disease protection to plants [186]. Based on the disease resistance mechanisms of endophytes, we can speculate that these enzymes may also improve salt tolerance in plants. Since many enzymes that hydrolyze fungal cell walls are encoded by single genes, these genes can be isolated and transferred into PGPB to create versatile salt-tolerant PGPB. ACC deaminase-related genes were transferred into salt-tolerant PGPB strains to regulate ethylene levels in halophytes and non-halophytes and improve salt tolerance [101]. Many bacterial endophytes can be cultured and can be directly applied to crops either by spraying, seed, or root inoculation [187]. A method for applying salt tolerant bacteria to agricultural production is to use bacterial capsules [188]. Bacterial capsules are polymer-coated outside of the bacteria, and the coating is positively charged and combines with a negatively charged cell wall to form a mixed capsule. It can be used to improve the survival and persistence of pathogenic bacteria on inoculated crops [188]. Bacterial capsules are non-toxic, durable, convenient, easy to store, and easy to apply. Rainfall or irrigation dissolves the capsule to slowly release the bacteria. Additionally, capsules can include a variety of beneficial bacteria with complementary activities to improve plant salt tolerance and reduce application costs [188].

### 6.3. Challenges in Applying Symbiotic Bacteria

The unique root microbiota of *Glycine soja* improve its adaptability to saline soil, but also benefit other non-halophytes such as *Sorghum dochna* and *Sesbania cannabina* [189]. Salt-tolerant bacteria isolated from the roots of the halophyte, *Arthrocnemum indicum*, enhanced the salt tolerance of peanut (*Arachis hypogaea*) seedlings [109], demonstrating that halophyte microbiota can be used on non-halophytes to improve salt tolerance. Can all halophyte microbiota promote salt tolerance? Are halophyte microbiota harmful to non-halophytes? Which PGPR and PGPEB species isolated from halophytes are most effective for improving salt tolerance? Which may play the greatest roles in promoting plant salt tolerance? What is the best application method for rhizobia: soaking, inoculation, or direct watering? All of these questions need to be investigated. Moreover, the vast majority of halophyte symbiotic bacteria have not been studied, and this information could help improve the salt tolerance of non-halophytes.

With increasing emphasis on environmental protection and agricultural sustainability, it is imperative to address the adverse effects of salt stress on plants in a cost-effective manner. Plant growth-promoting bacteria promote abiotic stress tolerance in crops, and studying their mechanisms will help improve crop growth under stress conditions. It is important to optimize bacterial strain combinations to address the abiotic stresses frequently encountered in crop production and to establish inoculation programs.

Moreover, clustered regularly interspaced short palindromic repeats (CRISPR)/CRISPR-associated protein 9 (Cas9) genome editing could be used to modify the stress response genes to improve plant stress tolerance. It is difficult to maintain bacterial populations after field inoculation, possibly due to the incompatibility between plants and bacteria, poor inoculation methods, or soil conditions, for example, saline areas are not suitable for bacterial proliferation, and pesticides may reduce bacterial survival. However, it is unclear whether the rapid and stable isolation of the halophytic symbiont and its application to non-halophytes will adversely affect some non-halophytes, or which side has more advantages and disadvantages. Moreover, plant growth-promoting bacteria function differently in different plants. In addition, current research on plant growth-promoting bacteria has mainly focused on the screening and chemical analysis of physiologically active substances, while their application in agricultural crops is not well studied.

Our understanding of the bacteria that promote plant growth has gradually improved. Environmentally friendly crop production practices have been established to reduce agricultural pollution from pesticides and fertilizers. These alternatives to traditional agricultural practices help reduce pollution and improve human health.

## Data Availability

Not applicable.
